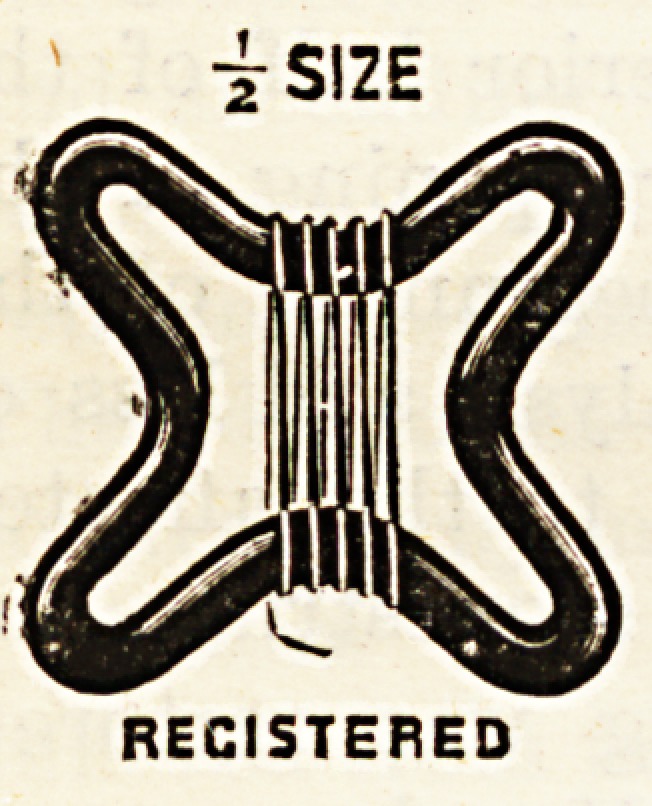# New Appliances and Things Medical

**Published:** 1896-11-28

**Authors:** 


### NEW APPLIANCES AND THINGS MEDICAL.

[We shall be glad to receive, at our Office, 28 & 29, Southampton Street, Strand, London, W.C., from the manufacturers, specimens of all new preparations and appliances which may be brought out from time to time.]

### NEW GLASS LIGATURE REEL.

(Messrs. REYNOLDS AND BRANSON, BRIGGATE, LEEDS.)

At the suggestion of a medical man, Messrs. Reynolds and Branson have made a new aseptic ligature reel, as shown in the illustration. It has the advantage over the usual solid reel, in that it obviates the necessity of prolonged boiling to sterilise the deeper layers of the silk. The reel is made of a glass rod, fashioned in the shape of a Maltese cross. It is made in various colours for different sized silk, so that they may be readily distinguished. The glass is toughened by the boiling, and does not readily break, and the reel is of such simple construction that the price is merely nominal.

½ SIZE

REGISTERED

### SPLINT PADDING.

(LIVERPOOL LINT COMPANY, MARK STREET MILLS, LIVERPOOL.)

This new dressing possesses many very obvious advantages. It consists of a thick layer of absorbent and antiseptic wadding, arranged between two layers of gauze. It is essentially a tidy and business-like dressing, lies far more evenly than ordinary layers of cotton wool, and being enclosed between layers of gauze does not cover the clothes of the surgeon or patient with the usual fluffy deposit, which is so particularly objectionable and difficult to remove. Where pressure is desirable a roll of the splint padding constitutes a reliable and permanent means of applying it, and similarly pressure can be avoided on any particular point, by counter pads of the same. Having made practical trial of this dressing, we recommend it to the notice of surgeons, not only in hospital practice, but for use among private patients.

### BORTHWICK'S BOUILLON.

(381, KINGSLAND ROAD, LONDON, N.)

We have received a sample of this well-known preparation, and our renewed acquaintance with it confirms the high opinion which we formed when it was first submitted to our notice. It is a very concentrated preparation, consisting of solid matter to considerably over 50 per cent., and these solids consist not only of extractives of meat, but also of peptones and albumoses—hence there are present in the bouillon not only stimulating but nutritive substances, which render the preparation eminently suited for the purpose of nourishing invalids and others whose strength has been lowered by wasting disease or mental worry. It is a distinctly agreeable beverage, and may be used alone or with all kinds of soups, stews, and hashes.

### SOLUBLE COCOA.

(TAYLOR BROTHERS, LONDON.)

This well-known cocoa has been submitted to us for notice. We find it of first rate quality, of pure constitution, free from excess of fat, highly soluble, and of a high degree of condensation. Half a teaspoonful is sufficient to make a large sized cup of excellent cocoa.

## Figures and Tables

**Figure f1:**